# The relation between stimulated salivary flow and the temporal consumption experience of a liquid oral nutritional supplement

**DOI:** 10.1016/j.appet.2021.105325

**Published:** 2021-11-01

**Authors:** S. Lester, K. Hurst, L. Cornacchia, M. Kleijn, C. Ayed, V. Dinu, M.A. Taylor, I. Fisk

**Affiliations:** a University of Nottingham, Division of Food, Nutrition and Dietetics, School of Biosciences Nottingham, UK; b University of Nottingham, National Institute for Health Research (NIHR) Nottingham Biomedical Research Centre, Division of Physiology, Pharmacology and Neuroscience, School of Life Sciences, University of Nottingham, Nottingham, UK; c Danone Nutricia Research, Uppsalalaan 12, 3584, CT, Utrecht, the Netherlands

**Keywords:** Saliva flow rate, Hyposalivation, Oral nutritional supplements (ONS), Temporal perception, Healthy ageing

## Abstract

Use of oral nutritional supplements (ONS) in undernourished patients has proven clinical benefits, but this can be hampered by low adherence due to poor experience of palatability. Many patients, particularly older patients, experience hyposalivation which can cause taste changes and reduce the enjoyment of foods. The aim of this study was to investigate differences in the temporal consumption experience (comprising sensory perception, in-mouth aroma release and subjective appetite) of a clinically relevant portion of ONS, for groups differing in saliva flow rates (SFR). The SFR (mL/min) of thirty healthy individuals was measured on three occasions. This data was used to categorise individuals into three groups using quartile analysis: low flow (LF) (0.3–0.6 mL/min, n = 5), medium flow (MF) (0.7–1.2 mL/min, n = 16) and high flow (HF) (1.3–1.8 mL/min, n = 9). Over the consumption of eight 15 mL sips of ONS, individuals rated their sensory perception and subjective appetite perception using line scales. Additionally, in-mouth aroma release was measured for each sip, using atmospheric pressure chemical ionisation (APCI). Compared with the MF and HF group, the LF group reported a significantly greater increase of mouth-drying over increased sips (p = 0.02). The LF group also experienced significantly higher aftertaste perception (p < 0.001), and more intense in-mouth aroma release (p = 0.015), compared with the HF group. These findings occurred concurrently with relatively lower hunger sensations in the LF and MF group. Many patients who are prescribed ONS likely experience reduced salivary flow rates. The unique sensory experiences of these individuals should be considered in order to optimise palatability and nutritional intake.

## Highlights

• A full portion of ONS was evaluated by three groups differing in saliva flow rates.
• A sensory profiling method captured perceptual differences over repeated sips.
• Mouth-drying built up most significantly for the low saliva flow group.
• Intensity of aftertaste and aroma release was highest in the low saliva flow group.
• Sensorial intensity of ONS may be associated with greater feelings of satiation.

## 1 Introduction

During food oral processing, the in-mouth interaction between saliva and food is essential for perceiving sensory properties (Chen & Engelen, 2012; Condelli, Dinnella, Cerone, Monteleone, & Bertuccioli, 2006; Dinnella, Recchia, Fia, Bertuccioli, & Monteleone, 2009; Fischer, Boulton, & Noble, 1994; Horne, Hayes, & Lawless, 2002; Mosca & Chen, 2017; Salles et al., 2010). Saliva is known to have a large influence on texture perception, for example, salivary enzymes facilitate the in-mouth digestion of macromolecules leading to a reduction in perceived thickness (Mosca & Chen, 2017). Some mouthfeel sensations, such as astringency, are suggested to be related to the type and quantity of salivary proteins such as proline-rich proteins (PRPs) (Dinnella et al., 2009; Dinnella, Recchia, Vincenzi, Tuorila, & Monteleone, 2010; Horne et al., 2002). The viscosity of saliva (a measure of a fluids resistance to flow) may also be crucial in driving food perception, for example, a low viscosity saliva is known to be more effective in clearance of food residue from the oral cavity (Chen & Engelen, 2012; Negoro et al., 2000).

Flavour perception is also largely dependent on saliva secretions. Hydrophilic tastants from foods diffuse through the salivary aqueous medium to reach taste buds on the tongue (Salles et al., 2010) and in-mouth aroma release is largely dependent on the volume and constituents within the saliva (Odake, Roozen, & Burger, 1998; Pagès-Hélary, Andriot, Guichard, & Canon, 2014; Ployon, Morzel, & Canon, 2017; Salles et al., 2010; Van Ruth, 2000; Yang, Galves, Racioni Goncalves, Chen, & Fisk, 2020). Volatile aroma compounds differ in chemical properties such as hydrophobicity, so the chemical nature of the volatile aroma compound, and the subsequent interactions with aqueous saliva and the salivary constituents, can determine the extent of their release into the gaseous olfactory-space (Odake et al., 1998; Ployon et al., 2017; Yang, Galves, RacioniGoncalves, Chen, & Fisk, 2020). These factors ultimately determine the type and extent of volatile aroma compounds that are perceived retro-nasally.

Individual saliva flow rates (SFR) and salivary composition varies across the course of a day (Dawes, 1975; Humphrey & Williamson, 2001), under exposure to stress (Jemmott et al., 1983), or in response to different food stimuli (Engelen, De Wijk, Prinz, Van Der ). There is also large variation between individuals, in quantity, properties, and concentrations of constituents within saliva secretions. Factors causing variations in healthy individuals include dietary intake (Dawes, 1970) smoking status (Edgar, O'Mullane, & Dawes, 2004; Rad, Kakoie, Brojeni, & Pourdamghan, 2010) and gender (Percival, Challacombe, & Marsh, 1994; Prodan et al., 2015).

Human ageing also is associated with chronic reductions in SFR and/or altered salivary compositions (Bossola et al., 2013; Edgar et al., 2004; Iwasaki et al., 2016; Narhi, Meurman, & Ainamo, 1999; Villa, Connell, & Abati, 2014) and the cause of these changes are multifaceted. Older adults are more susceptible to dehydration, as thirst signalling mechanisms are impeded in older age (Schols, Groot, Cammen, & Olde), and dehydration has been proposed as one of the most important factors contributing to salivary hypofunction (Dawes, 1970; Edgar et al., 2004; Narhi et al., 1999). SFR and salivary compositions are also known to be strongly influenced by certain age-related diseases such as Parkinson's disease, cancer, stroke and diabetes (Edgar et al., 2004), in addition to the medications and treatments used to treat them (Edgar et al., 2004; Villa et al., 2014). For example, patients with cancer frequently experience long-term reductions in SFR or compositional changes due to radiotherapy treatment, particularly when administered in the head and neck region (Henson, Eisbruch, d’Hondt, & Ship, 1999; Laheij et al., 2015; Rogus-Pulia et al., 2016; Villa et al., 2014).

It is not surprising therefore that many patients, particularly those of an older age, receive a clinical diagnosis of hyposalivation (Narhi et al., 1999; Villa et al., 2014). Hyposalivation is defined as a measurable decrease in the amount of saliva in the mouth, and objectively defined as a stimulated flow rate of ≤0.5 mL/min (Edgar et al., 2004; Iwasaki et al., 2016; Narhi et al., 1999; Nederfors, 2000; Villa et al., 2014).

Patients with hyposalivation frequently complain of taste changes (Villa et al., 2014) so it could be hypothesised that salivary variations may be a contributing cause. Furthermore, sensory perception occurs concurrently alongside physiological phenomena that regulate appetite establishing a sensorial feedback mechanism that notifies the consumer about the nutritional and satiating properties of foods (Gibson & Brunstrom, 2007; Ramaekers et al., 2014). Consequently, it has been proposed that hyposalivation may be a risk factor for reduced nutritional intake (Muñoz-González, Feron, & Canon, 2018) and could potentially contribute to undernutrition and involuntary weight loss (Iwasaki et al., 2016; Sullivan, Martin, Flaxman, & Hagen, 1993).

For individuals who are undernourished or at risk of a nutritional deficiency, oral nutritional supplements (ONS) are often prescribed to supplement or replace the oral nutritional intake. ONS are usually liquids, hence less satiating than nutritionally equivalent solids (Zijlstra, Mars, De Wijk, Westerterp-Plantenga, & De ) and easy to consume by those with poor dentition. Although the clinical effectiveness of ONS has been proven (Stratton & Elia, 2007), patients must consume the prescribed volume in order to gain the nutritional benefits. However, adherence to the full prescription is known to be challenging and patients frequently terminate consumption before the prescribed volume is consumed (Gosney, 2003). Poor palatability has been proposed as a key factor limiting sufficient intake of ONS (Den Boer, Boesveldt, & Lawlor, 2019; Kennedy, Law, Methven, Mottram, & Gosney, 2010). Food sensory properties known to be important to the palatability and intake of dairy-based ONS are thickness (Den Boer et al., 2019), sweetness (Den Boer et al., 2019; Kennedy et al., 2010; Methven et al., 2010), off-tastes (Methven et al., 2010), aftertaste (Regan, O'Neill, Hutchings, & O'Riordan, 2019) and mouth-feel effects, such as mouth-drying and mouth-coating (Methven et al., 2010; Thomas, Van Der Stelt, Prokop, Lawlor, & Schlich, 2016; Withers, Gosney, & Methven, 2013). The undesirable mouth-drying phenomenon is known to build up over repeated sips of a consumed portion (Methven et al., 2010). For ONS to have the greatest clinical success, they must be palatable to the consumer to facilitate adequate intake. Considering that patients frequently experience hyposalivation, it is important to understand how variations in saliva flow rate and composition influence the sensory perception of ONS.

We hypothesise that SFR and saliva composition may be associated with the consumption experience of ONS. Subsequently, findings may support our understanding of factors which potentially lead to early termination of ONS intake. As food experiences are known to change over repeated intakes, our overarching aim was to investigate differences in the temporal consumption experience (comprising sensory perception, in-mouth aroma release and subjective appetite) of a clinically relevant portion of ONS, for groups differing in SFR, in which repeated measurements were made between sips. Specific salivary parameters (such as saliva protein content and saliva viscosity) were also characterised for each group, as it was hypothesised that these may be crucial in our understanding of potential group differences. Unravelling the link between saliva composition and consumption experience is a fundamental step towards the design of nutritional formulations adapted to the specific consumer need.

## 2 Materials and methods

This study was approved by Faculty of Medicine and Health Sciences Research Ethics Committee at the University of Nottingham (Reference No. 207–1902).

### 2.1 Participants

The study was conducted in the Food Flavour Laboratory on Sutton Bonington Campus at The University of Nottingham. Forty healthy adults were recruited to take part in the study via an email invitation. We chose to recruit healthy individuals with differing saliva rates, rather than patients, to limit the additional influences of medication and disease on sensory perception and appetite. Inclusion criteria were: aged between 18 and 40 years, self-reported health, healthy BMI within the range 18.5–24.9 kg/m^2^, non-smoking and complete dentition. Exclusion criteria included food allergies or intolerances, physical or mental health problems, poor dental health, medication use (excluding oral contraceptives), pregnancy and lactation, and known sensory impairments in taste and smell.

#### 2.1.1 Screening

All potential participants were electronically provided with information about the study, and then invited to a screening visit in order to assess their eligibility. On this screening visit, the study was explained to the participants and they were invited to complete a questionnaire containing health, lifestyle and demographic questions. Height was measured to the nearest 0.1 cm using a stadiometer (Seca®, Germany). Body weight was measured using an electronic scale to the nearest 0.1 kg (Seca®, Germany) whilst participants were wearing light clothing with no shoes and an empty bladder. BMI was calculated from their height and weight as kg/m^2^. Ten participants did not fit the criteria (their calculated BMI was outside the healthy range) and were therefore not invited to take part. Thirty participants met the inclusion criteria, so they were invited to take part in the study and informed, written consent was obtained.

### 2.2 Overview of study design

Participants attended three study sessions in total, which were one week apart, and occurred at the same time of day for each individual (between 9am and 6pm). Participants were required to not eat or drink for 2 h prior to each session and not exercise strenuously or drink alcohol for 24 h prior to each session.

At Session 1 (15 min), which immediately followed the screening session, participants were required to provide a stimulated saliva sample.

At Session 2 (1 h), each participant provided their second stimulated saliva sample and following this underwent a 30-min training session on sensory attributes and to standardise drinking behaviour.

At Session 3 (1 h), each participant provided their third stimulated saliva sample (15 min) and following a short break, completed the ONS consumption study (2.3).

#### 2.2.1 Protocol for saliva collection and determination of flow rate

Stimulated saliva was collected by asking individuals to chew continuously on a clean square of Parafilm® for 15 min. Every time the individual felt they needed to swallow they were asked to expectorate their saliva into a sterile polypropylene graduated collection tube. Once collected, the weight of saliva (g) was determined by weighing the collection tube before and after saliva collection. In line with previous research (Norton, Lignou, Bull, Gosney, & Methven, 2020) saliva volume (mL) was determined with the assumption that 1g of saliva is equal to 1 mL, and the stimulated salivary flow rate (SFR) calculated (mL/min).

The saliva was immediately separated into individual 1 mL aliquots for further analysis. To prevent degradation during viscosity analysis (2.2.2) a protease inhibitor (2 μL protease inhibitor cocktail, Sigma Aldrich®) was added to the aliquots. The aliquots for the protein measurements (2.2.3) were immediately frozen at −80 °C.

#### 2.2.2 Rheological analyses

Salivary viscosity was measured immediately after collection.

A Modular Compact Cone-Plate Rheometer MCR 302 (Anton Paar GmbH, Germany) was used. The cone used was a CP50-2/TG with diameter 49.957 mm, angle 2.006°, truncation 208 μm. Analysis was carried out at 37 °C. 5 points per decade were used for 3 decades with shear rate increasing logarithmically from 1 to 1000 s^−1^. A total of 15 points were made, 1 point per minute. Rheoplus analysis software (Anton Paar GmbH, Germany) was used. The sample volume was 1.0 mL.

The viscosity at a sheer rate of 50s^−1^ was used in the data analysis as this closely represents the forces within the oral cavity during the movement of liquids (Chen & Engelen, 2012).

#### 2.2.3 Protein concentration and α-amylase activity

Saliva samples were kept frozen at −80 °C for a period no longer than 24 h. Once removed from the freezer, the saliva samples were defrosted at room temperature for a period no longer than 5 min and then underwent a gentle centrifugation (1500 g for 15 min) to remove large cellular debris. Total protein content (TPC, mg/mL) was determined by using a colorimetric assay based on bicinchoninic acid (Pierce™ BCA Protein Assay Kit). Protein Secretion Rate (PSR, mg/min) was determined by multiplying the protein concentration by the saliva flow rate. The salivary activity of α-amylase (AA, U/mL) was determined using a colorimetric assay based on 2-chloro-p-nitrophenol linked with maltotriose (Salimetrics® Salivary Alpha-Amylase Assay Kit).

### 2.3 ONS consumption study session protocol and procedures

Prior to the participants entering the lab, one full portion of a banana flavoured ONS (125 mL, Fortisip Compact Energy, Nutricia B.V., Zoetermeer, The Netherlands) was separated into 8 individual sips (15.6 mL per sip) by the experimenter. The total portion of ONS contained 12 g protein, 11.6 g fat and 37.1 g carbohydrate, comprising 300 kcal. The temperature of the sips was controlled using a water bath set to 20 °C.

On arrival at the lab, a plastic tube was inserted into one of the participant's nostrils to sample their expired air. This tube was connected to the atmospheric pressure chemical ionisation (APCI) apparatus and allowed measurement of continuous in-mouth aroma release whilst the sips of ONS were consumed. Participants consumed the sips from a standard unbranded ONS bottle using a straw. Instructions were provided on an iPad (Apple, UK) using Compusense® and each total consumption event (8 sips) lasted approximately 15 min. Prior to the study session, in an effort to standardise drinking behaviour, participants were instructed and trained to consume each sip in a standardised way: consume as much of each pre-measured sip (15.6 mL) from the bottle as possible, control oral transit time to 1 s by counting and swallow in one mouthful (not multiple swallows).

**Fig. 1:**
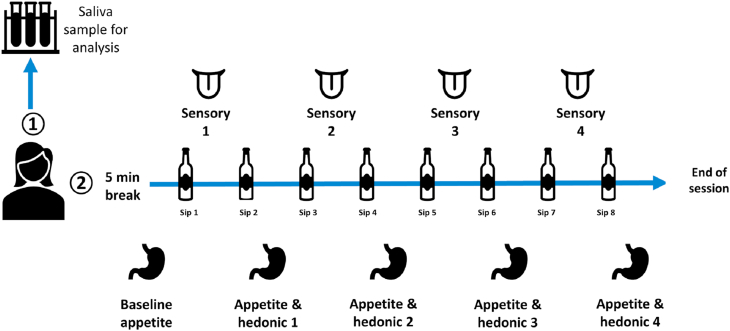
Outline of the study session whereby participants provided stimulated saliva sample (1) and proceeded to consume 8 sips of ONS whilst alternately rating sensory and appetite variables (2).

Participants rated their subjective perception of sensory attributes and appetite during the consumption of the 8 sips (See Fig. 1. This data was collected electronically using Compusense®. Participants first gave a baseline appetite rating at sip 0 and following this, sensory and appetite ratings were made alternately between sips. Sensory ratings were thus made after sips 1, 3, 5 and 7 whereas hedonic perception and subjective appetite ratings were made after sips 2, 4, 6 and 8. A compulsory 1-min break was given between each rating.

#### 2.3.1 Sensory perception

Perceived intensity of sensory attributes was measured using an unstructured line scale with appropriate anchors. The methodology used to collect sensory data was based on the sequential profiling method developed by Methven et al. (2010), which permits sensory profiling of a number of attributes over the repeated consumption of ONS aliquots. The original method involves rating immediately after each aliquot, followed by another rating at 30 s and 60 s. In the current study, instead of making 3 measurements per aliquot, the method was simplified to a single measurement taken immediately after each sip. This was to reduce participant fatigue from repeated tasting and scoring.

Four important sensory attributes to describe ONS were chosen from the literature (Methven et al., 2010; Regan et al., 2019; Thomas et al., 2016). These attributes were Sweetness, Mouth-drying, Mouth-coating and Aftertaste. In order to measure intensity of flavour, as the ONS was banana flavoured, Banana Flavour was also chosen to be an attribute. The description of these attributes is given in Table 1. The order of presentation of the first four attributes were randomised between individuals and between sips, but the attribute aftertaste was anchored to be asked last.

**Table 1:** Sensory attributes including the description and physical references used to train participants to recognise sensations.

Attribute	Description	Reference
Sweetness	The intensity of sweet flavour	Semi-skimmed milk with 5% sucrose
Banana flavour	The intensity of banana flavour	Semi-skimmed milk with banana flavourings
Mouth-drying	The intensity of a drying sensation in the mouth	Milk containing whey protein after being heated to 70 °C for 20 min
Mouth coating	The intensity of milk clinging to the surface of the mouth	Full fat cream
Aftertaste	The intensity of flavour lingering in the mouth	No physical reference but participants asked to consider both sweetness and banana flavour

##### 2.3.1.1 Training on sensory attributes

Prior to taking part, participants underwent a short training session to ensure they understood the meaning of each sensory attribute. Participants were provided with written definitions of each attribute and asked to read through them. After confirming they understood the meaning, participants were provided with physical references for each attribute and asked to taste them (Table 1). For aftertaste, no reference was provided but participants were asked to consider *any* lingering flavour in the mouth, considering both the attributes Sweetness and Banana flavour. Participants were also shown an example of the line scales.

#### 2.3.2 Hedonic perception and subjective appetite sensations

In-line with the sensory scales (2.3.1), hedonic perception and subjective appetite sensations were made on unstructured line scale with appropriate anchors. After providing their initial baseline appetite rating (sip 0 before any ONS consumption) participants were asked to rate their hedonic perception of ONS and subjective appetite (after sips 2, 4, 6 and 8) using a series of questions (Table 2). A baseline was not collected for the hedonic questions 1 or 6. Questions were not randomised and always anchored in the specific order shown.

**Table 2:** Appetite and hedonic questions asked at sips 3, 5, 7 and 9. Baseline scores (sip 0) were also collected for questions 2–5.

Hedonic and appetite questions asked
1. How pleasant would you rate the beverage?
2. How hungry do you feel right now?
3. How full do you feel right now?
4. How much do you think you could eat right now?
5. How strong is your desire to eat right now?
6. How strong is your desire to drink more of the beverage?

#### 2.3.3 In-mouth aroma release

For the period which ONS was being consumed, the expired air of each participant was sampled at a flow rate of 45 mL/min^−1^, through a plastic tube inserted into the exterior opening of the nostril. A MS-Nose™ (Micromass, Manchester, UK) interface and a Quattro Ultima mass spectrometer (Waters Corporation, Milford, MA) was used to monitor the in-mouth release of four aroma molecules in real-time. The molecules were isoamyl acetate (*m/z* 130), isoamyl propionate (*m/z* 144), isoamyl isovalerate (*m/z* 172), ethyl butyrate (*m/z* 116). These flavour molecules have previously been identified as significant aroma-active contributors to the flavour of banana flavour ONS (data not yet published).

### 2.4 Statistical analysis

All statistical analysis was conducted using GraphPad Prism version 8.1.2 (GraphPad Software, San Diego, CA, USA).

For each individual, the mean SFR of the three biological replicates was calculated. Quartile analysis of the mean SFR values was used in order to define three groups (Condelli et al., 2006; Dinnella et al., 2010; Shen, Kennedy, & Methven, 2017). Individuals with a mean SFR less than the first quartile (Q1) determined the low salivary flow rate group. Individuals with an SFR the same as or greater than Q1, but less than the third quartile (Q3), defined the medium salivary flow group. Individuals with SFR the same as or greater than Q3 defined the high salivary flow rate group.

For each salivary variable (viscosity, TPC, PSR and AA), the individual mean of the three biological replicates was calculated in addition to group means and standard error. Linear regression assessed relationships between salivary variables and Pearson correlation coefficient was used to determine whether correlations were statistically significant (p ≤ 0.05). Statistically significant differences between group salivary variables was assessed with one-way ANOVA (p ≤ 0.05).

For each sensory, hedonic and appetite variable, the group mean and standard error values were calculated. A one-way ANOVA was used to assess whether statistically significant differences existed between groups for baseline appetite scores. Subjective appetite scores (Hunger, Fullness, Desire to eat, Prospective Consumption) were processed to generate Change From Baseline (CFB) scores by subtracting baseline scores from each score measured at Sips 2, 4, 6 and 8. Differences between groups, in sensory ratings and appetite CFB ratings, were analysed by Mixed Models ANOVA, with Group as a between-subjects factor and Sip as a within-subjects factor. Sphericity was not assumed, therefore Greenhouse-Geisser corrections were used to adjust *df* and p-values of within-subject factors.

For in-mouth aroma release data, the total ion count (TIC) was used for statistical analysis. Three release parameters were extracted from each ‘swallow breath curve’ generated for each individual sip: maximum intensity (Imax), time to reach maximum intensity (Tmax) and area under the curve (AUC). Group mean and standard error values were calculated. Release parameters were analysed by two-way ANOVA with Sip as a within-subjects factor and Group as a between-subjects factor. Where any statistically significant effects were found (p ≥ 0.05), *post hoc* pairwise comparisons were conducted (Tukey's test).

## 3 Results

30 participants completed the study, 9 males and 21 females, between the ages of 20 and 45 years. Mean characteristics of participants can be found in Table 3.

**Table 3:** Demographic information for each group classified by saliva flow rate.

Group	n	Mean SFR (mL/min, ± SD)	Mean age (years, ± SD)	Mean BMI (kg/m^2^, ±SD)	Male: Female
LF group	5	0.4 (0.1)	27 (3)	22.4 (2.7)	1:4
MF group	16	1.0 (0.2)	29 (7)	22.5 (2.1)	7:9
HF group	9	1.5 (0.2)	27 (4)	23.2 (1.9)	1:8
Average (mean)	30	1.0 (0.4)	28 (6)	22.7 (2.0)	9:21

### 3.1 Participant grouping by salivary flow rate

**Fig. 2:**
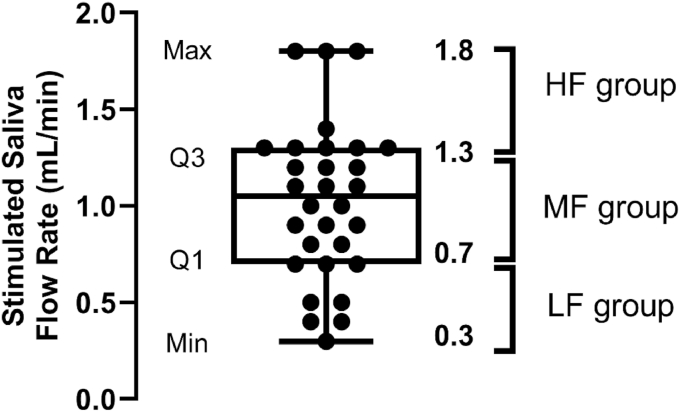
Quartiles of mean stimulated saliva flow rates for each participant. The minimum value (Min), Quartile 1 (Q1), Quartile 3 (Q3) and the maximum value (Max) are indicated.

Mean stimulated salivary flow rates of all participants (n = 30) ranged from 0.3 mL/min to 1.8 mL/min (mean 1.0 mL/min). Individuals were categorized into the low flow rate group (LF group) if their flow rate was < Q1 (n = 5, 0.3–0.6 mL/min), medium flow rate group (MF group) if their flow rate ≥ Q1 or < Q3 (n = 16, 0.7 −1.2 mL/min), and high flow rate group (HF group) if their flow rate was ≥ Q3 (n = 9, 1.3–1.8 mL/min) (Fig. 2).

### 3.2 Characterisation of salivary constituents

Table 4 reports correlations between mean salivary variables for pooled data. A significant negative correlation was observed between SFR and viscosity (p = 0.04), indicating that low-flow rate saliva is more viscous. A significant positive correlation was observed between viscosity and TPC (p = 0.02), indicating that a low viscosity saliva contains a higher concentration of proteins. As expected, a significant strong positive correlation was observed between SRF and PSR (p < 0.01), indicating that individuals with higher SFR secrete more proteins over time. AA was significantly positively correlated to TPC and PSR (p < 0.01).

**Table 4:** Correlations between salivary variables: Saliva Flow Rate (SFR), Saliva Viscosity, Total Protein Content (TPC), Protein Secretion Rate (PSR) and α-amylase activity (AA). Significant relationships (p ≤ 0.05) are highlighted in bold text.

	SFR	Viscosity	TPC	PSR	AA
SFR					
Viscosity	**r = -0.376** **p = 0.04**				
TPC	r = −0.235 p = 0.21	**r = 0.441** **p = 0.02**			
PSR	**r = 0.734** **p < 0.01**	r = −0.109 p = 0.57	**r = 0.431** **p = 0.02**		
AA	r = 0.168 p = 0.37	r = −0.026 p = 0.89	**r = 0.529** **p < 0.01**	**r = 0.511** **p < 0.01**	

**Fig. 3:**
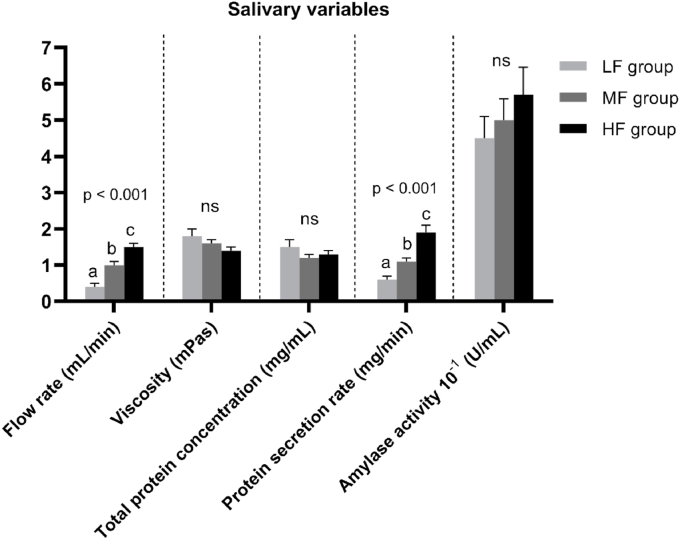
Mean values (±standard error) for each salivary variable (Flow rate (mL/min), Viscosity (mPas), total protein concentration (mg/mL), protein secretion rate (mg/min) and amylase activity 10^−1^ (U/mL) for the Low flow (LF), Medium flow (MF) and High flow (HF) groups. Significant group differences were analysed by one-way ANOVA (p ≤ 0.05). Means with statistical group difference are indicated by different letters, ns = not significant (Tukey post-hoc test).

When analysed by ANOVA (Fig. 3), SFR was found to be significantly different between groups (p < 0.001) and pairwise comparisons revealed that the LF group had a SFR significantly lower than the MF (p < 0.001) and HF group (p < 0.001), the MF group also had a SFR significantly lower than the HF group (p < 0.001).

From observing Table 4 we can see that the LF group had greater saliva viscosity and TPC, but lower PSR and AA, when compared with the MF and HF saliva groups. When analysed by ANOVA, PSR (mg/mL) was significantly different between groups and pairwise comparisons revealed that PSR was significantly lower in the LF group, compared with the MF (p = 0.03) and HF (p < 0.001) groups.

### 3.3 Temporal experience of ONS

#### 3.3.1 Sensory perception

**Fig. 4:**
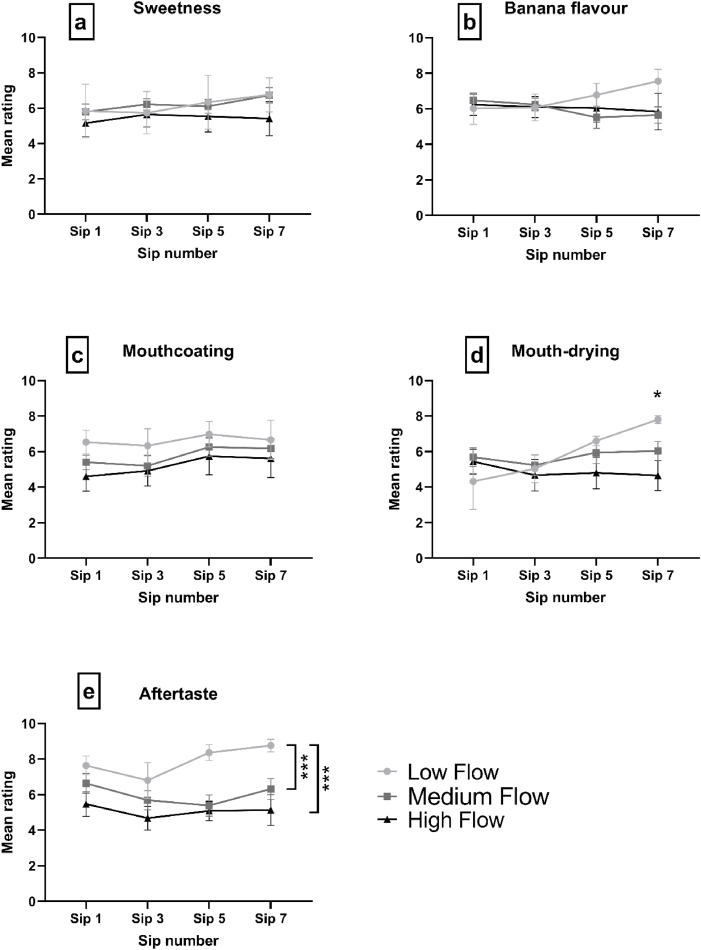
Mean group intensity (±standard error) of sensory perception, as rated by each group differing in saliva flow rate, for each separate sensory attribute (a–e) over the consumption of four sips (sip 1, 3 5 and 7) of ONS. Main effects and simple main effects are indicated by *p < 0.05, **p < 0.01, ***p < 0.001.

Immediately following consumption of sips 1, 3, 5 and 7 of the ONS, participants rated the intensity of five sensory attributes. Results are reported in Fig. 4.

##### 3.3.1.1 Sweetness

Fig. 4a shows that all groups reported similar sweetness intensity over sips. No significant interaction effects were found between group and sip [F = (6, 81) = 0.359, p = 0.902]. No significant main effects of group [F = (2, 27) = 0.570, p = 0.572] or sip [F = (2.33, 62.92) = 1.155, p = 0.327] were found for sweetness.

##### 3.3.1.2 Banana flavour

Fig. 4b shows that all groups reported similar banana flavour intensity ratings over sips, though the LF group reported a small increase in intensity at Sip 7. Statistical analysis revealed that there were also no significant interaction effects between group and sip [F = (6, 81) = 1.21, p = 0.310]. There were no significant main effects of group [F = (2, 27) = 0.335, p = 0.718] or sip [F = (1.94, 52.33) = 0.134, p = 0.869].

##### 3.3.1.3 Mouthcoating

There were no significant interaction effects between group and sip [F = (6, 81) = 0.174, p = 0.983]. From visually inspecting Fig. 4c, it can be seen that the LF group reported higher mouthcoating intensity than the MF and HF groups over sips, though this effect was not statistically significant [F = (2, 27) = 0.806, p = 0.457]. No significant main effects of sip were found [F = (2.45, 66.13) = 2.17, p = 0.112].

##### 3.3.1.4 Mouth-drying

From visually inspecting Fig. 4d, the MF and HF group show little change in perception of mouth-drying intensity over increasing sip numbers. This contrasts with the LF group, where an increase in mouth-drying intensity is reported from sip 1 to sip 7, by 3.4 points on the scale. There was a significant interaction between group and sip [F = (6, 81) = 2.594, p = 0.024]. Pairwise comparisons of the simple effects of groups within each time point revealed that, at sip 7, the LF group reported significantly higher mouth-drying intensity than the MF (p = 0.027) and HF group (p = 0.017).

##### 3.3.1.5 Aftertaste

From visually inspecting Fig. 4e, it is apparent that the LF group reported higher aftertaste intensity, particularly for the latter sips (Sip 5 and Sip 7). There were no interaction effects between group and sip [F = (6, 81) = 1.210, p = 0.310]. A significant main effect of group was found [F = (2, 27) = 4.009, p = 0.030] and pairwise comparisons revealed that the LF group rated significantly higher intensity of aftertaste compared with the MF group (p < 0.001) and the HF group (p < 0.001). No significant main effects of sip were found [F = (2.331, 62.93) = 2.738, p = 0.064].

#### 3.3.2 Hedonic perception and subjective appetite sensations

Immediately following consumption of sips 2, 4, 6 and 8 of the ONS, participants rated hedonic perception (comprising pleasantness and desire to drink more) along with subjective appetite sensations (hunger, fullness, desire to eat and prospective consumption).

For the appetite variables, as analysed by one-way ANOVA, no significant difference in baseline values between groups were found and change from baseline (CFB) values were calculated for use in subsequent ANOVA analyses.

##### 3.3.2.1 Pleasantness of beverage

**Fig. 5:**
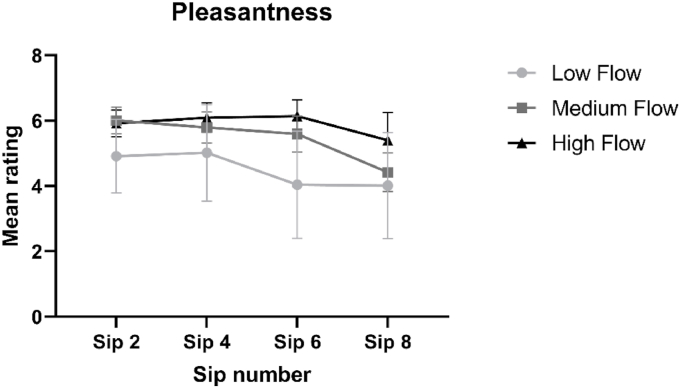
Mean group subjective ratings of pleasantness (±standard error) of sips 2, 4, 6 and 8 of the ONS, as rated by each group differing in saliva flow rate.

From visual inspection of Fig. 5, the data suggests that the LF group rated pleasantness lower compared with the MF and HF group. No significant interaction effects were found [F = (6, 81) = 0.547, p = 0.771] and likewise no significant main effect of group was found [F = (2, 27) = 0.8942, p = 0.421]. A borderline insignificant main effect of sip was found [F = (1.964, 53.03) = 3.049, p = 0.057]. Pairwise comparisons revealed that the participants perceived the ONS as increasingly less pleasant during consumption (for pairwise comparisons see Appendix E).

##### 3.3.2.2 Desire to drink more of beverage

**Fig. 6:**
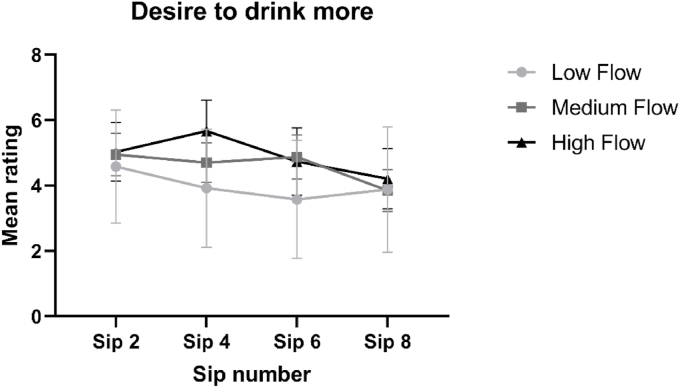
Mean group ratings of Desire to drink more of the ONS (±standard error), as rated by each group differing in saliva flow rate, over the consumption of four sips (sip 2, 4, 6 and 8) of ONS.

Fig. 6 shows there is little difference between groups in their desire to drink more of the ONS over increasing sips, though all groups show a decline in desire over increasing sips. No interaction effects were found between group and sip [F = (6, 81) = 1.053, p = 0.398]. A significant main effect of sip was found [F = (2.231, 60.23) = 3.098, p = 0.047] and pairwise comparisons revealed that participants desire to drink more of the beverage decreased significantly during consumption (for pairwise comparisons see Appendix E). No significant main effect of group was found [F = (2, 27) = 0.180, p = 0.836].

##### 3.3.2.3 Hunger

**Fig. 7:**
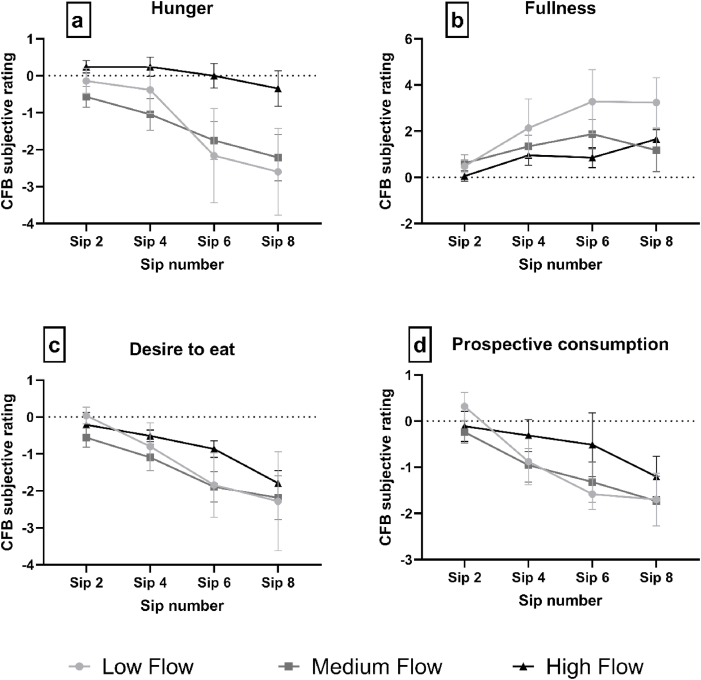
Mean group Change From Baseline (CFB, ± standard error) appetite scores, as rated by each group differing in saliva flow rate, for each appetite variable (a–e) over the consumption of four sips (sip 2, 4, 6 and 8) of ONS.

From visually inspecting Fig. 7a, we can see that the LF and MF group decrease in hunger ratings over increased sips of ONS, whereas the HF group show little change in their hunger ratings. No significant interaction effects between sip and group were found [F = (6, 81) = 1.822, p = 0.105]. A significant main effect of sip was found [F = (1.450, 39.16) = 14.17, p < 0.001], and pairwise comparisons revealed a suppressive effect of ONS on hunger during consumption (for pairwise comparisons see Appendix E). There was a borderline insignificant effect of group [F = (2, 27) = 2.764, p = 0.081] and pairwise comparisons revealed that the HF group has significantly higher CFB values, compared with the MF and LF groups (p < 0.05), indicating relatively less decrease in hunger during ONS consumption.

##### 3.3.2.4 Fullness

From visually inspecting Fig. 7b we can see that all groups show an increase in fullness ratings over increasing sips of ONS. No interaction effects between sip and group were found [F = (6, 81) = 1.160, p = 0.336]. A significant main effect of sip was found [F = (2.026, 54.71) = 6.247, p = 0.004] and pairwise comparisons revealed a significant increase in subjective fullness ratings during ONS consumption (for pairwise comparisons see Appendix E). No significant main effect of group was found [F = (2, 27) = 1.029, p = 0.371].

##### 3.3.2.5 Desire to eat

Fig. 7c shows that all groups decline in their reported desire to eat over increasing sips of ONS. No significant interaction effects between sip and group were found [F = (6, 81) = 0.620, p = 0.714]. A significant main effect of sip was found [F = (1.506, 40.65) = 18.10, p < 0.001] and pairwise comparisons revealed that participants reported desire to eat decreased significantly during consumption of the ONS (for pairwise comparisons see Appendix E). There was no significant main effect of group [F = (2, 27) = 0.5843, p = 0.564].

##### 3.3.2.6 Prospective consumption

Fig. 7d shows that all groups reported a decline in the perception of the amount they could eat over increasing sips. There does not appear to be any major differences between groups, though the HF group rated marginally higher ratings at sips 4, 6 and 8, compared with the MF and LF groups. No significant interaction effects between sip and group were found [F = (6, 81) = 0.946, p = 0.467]. A significant main effect of sip was found [F = (1.957, 52.84) = 15.06, p < 0.001] and pairwise comparisons revealed that participants’ perception of the amount which they could eat decreased significantly over increasing sips (see Appendix E for pairwise comparisons). No significant main effect of group was found [F = (2, 27) = 0.4804, p = 0.624].

#### 3.3.3 In-mouth aroma release

**Fig. 8:**
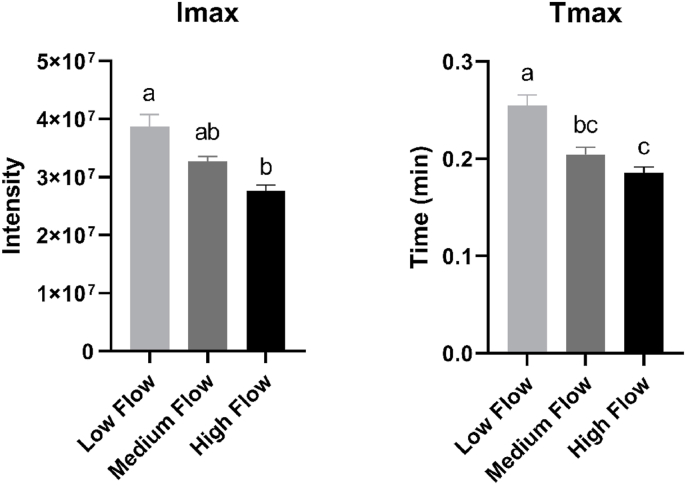
Mean Imax and Tmax values, averaged across eight sips of ONS, for each group. Means with statistical group difference are indicated by different letters, ns = not significant (Tukey post-hoc test).

Total ion count (TIC) was used for data analysis. Three release parameters were extracted from each ‘swallow breath curve’ generated for each individual sip: maximum intensity (Imax), time to reach maximum intensity (Tmax) and area under the curve (AUC). No significant Sip*Group interaction effects were found for I max [F = (14, 216) = 0.3101, p = 0.992], T max [F = (14, 216) = 0.6735, p = 0.799] or AUC [F = (14, 216) = 0.1951, p = 0.999]. A significant main effect of group was found for I max [F = (2, 216) = 4.025, p = 0.019] and T max [F = (2, 216) = 7.445, p < 0.001] (Fig. 8). Pairwise comparisons revealed that the LF group had significantly higher intensity of aroma release (Imax) than the HF group (p = 0.015). Pairwise comparisons also revealed that the LF group took significantly longer to reach maximum intensity (T max) compared with the MF (p = 0.008) and HF group (p < 0.001). No significant differences were found between group AUC values [F = (2, 216) = 0.6022, p = 0.549]. No significant main effects of Sip were found for I max [F = (7, 216) = 0.3502, p = 0.93], T max [F = (7, 216) = 0.2992, p = 0.954] or AUC [F = (7, 216) = 0.3478, p = 0.931].

## 4 Discussion

The aim of this study was to investigate the temporal consumption experience (comprising sensory perception, in-mouth aroma release and subjective appetite) of a clinically relevant portion of ONS, for groups of healthy individuals differing in their stimulated saliva flow rate (SFR). Specific salivary parameters, such as protein content and saliva viscosity, were characterised as it was hypothesised that these may be relevant in our understanding of potential group differences.

To classify individuals into groups, SFR was measured on three occasions, at the same time of day for each individual. Mean SFR of participants, averaged across the three replicates, were found to range from 0.3 to 1.8 mL/min. The three groups differed significantly in their SFR. Normal SFR are known to be within the range 1.5–2 mL/min (Villa et al., 2014), and a value ≤ 0.5 mL/min can be used as a cut-off value for diagnosis of hyposalivation (Edgar et al., 2004; Iwasaki et al., 2016; Narhi et al., 1999; Nederfors, 2000; Villa et al., 2014). We were therefore confident that individuals in the LF group, with a mean SFR of 0.4 mL/min (range 0.3–0.6 mL/min), had SFR closely similar to individuals with hyposalivation.

Significant differences between groups in perception of mouth-drying over repeated sips of ONS were found. The LF group increased considerably in perception of mouth-drying over increased sips of ONS, whereas the MF and HF groups remained relatively constant in their perception of mouth-drying (Fig. 4d). This ‘build up’ of mouth-drying has been found previously during ONS consumption by trained sensory panellists. Methven et al. (2010) found mouth-drying to increase by around 30 points, on the 0–100 point scale, over the consumption of eight sequential 5 mL aliquots of an ONS; though, it must be noted that the contribution of participant saliva to mouth-drying perception was not measured. More recently, Norton et al. (2020) found no significant differences in perception of mouth-drying, after consumption of a whey protein beverage, between groups differing in SFR. Though, Norton et al. (2020) measured mouth-drying after consumption of a single portion, and therefore it is likely that significant perceptual group differences were not able to ‘build up’ over repeated consumption events. We propose that individuals with low saliva flow rates are most susceptible to a greater ‘build up’ of mouth-drying over multiple intakes. For patients who experience hyposalivation, consumption of ONS could aggravate mouth dryness and may contribute to premature termination of ONS intake. Subsequently, to mitigate this build-up of undesirable mouthfeel sensations, it may be advantageous to provide patients with an acute saliva stimulating intervention alongside the administration of ONS. This may lead to improvements in adherence to ONS and a greater nutritional intake.

As far as the present authors are aware, the relationship between low SFR and greater mouth-drying perception has not been found previously. Though, there is evidence to show that low SFR is associated with greater astringency sensations (Condelli et al., 2006; Fischer et al., 1994; Horne et al., 2002; Ishikawa & Noble, 1995) of which, the mouth-drying sensation may be caused by a similar mechanism. However, salivary volume does not seem to account by itself for differences in astringency perception (Dinnella et al., 2010; Horne et al., 2002) and may be more strongly related to variabilities in saliva composition, as described below.

Astringency sensations are thought to be caused by a disruption of the lubricating pellicle coating the oral tissues, by astringent food compounds such as tannins, leading to greater oral friction (Breslin, Gilmore, Beauchamp, & Green, 1993; Prinz & Lucas, 2008) and ultimately a subjective dry, puckering sensation in the mouth. It has been suggested that salivary proline-rich proteins (PRPs), which contribute over 70% of proteins in stimulated saliva (Dinnella et al., 2009; Kauffman & Keller, 1979), may play a protective role, due to their ability to strongly bind and eliminate astringent compounds from the oral cavity (Dinnella et al., 2009; Horne et al., 2002). In the current study, the LF group had a significantly lower rate of protein output (0.6 mg/min) compared with the HF group (1.90 mg/min), due to the higher stimulated flowrates. Therefore, we propose that in between sips, the HF group was able to efficiently replenish the proteins within the oral cavity, providing protection to their oral environment. In contrast, the LF group was relatively less able to replenish proteins between sips of ONS. Therefore, the LF group had less protection of the lubricating pellicle over time and a gradual delubrication of oral tissues occurred, leading to the greater drying sensation. This is supported by Dinnella et al. (2009) who observed that a group who is less able to rapidly restore their protein contents in the mouth perceived significantly higher astringency sensations. As an expansion of this finding, Dinnella et al. (2010) found groups which were less able to restore protein contents, experienced higher build-up of astringency sensation over repeated samples.

Alternatively, it has been proposed that the mechanism behind dairy-originating mouth drying may not be the same as with true astringency, but could alternatively be explained by mucoadhesion of dairy proteins within the oral cavity (Cook, Bull, Methven, Parker, & Khutoryanskiy, 2017; Norton et al., 2020; Withers et al., 2013). In the context of food proteins, such as casein and whey, mucoadhesion is the binding of proteins to the mucosa surrounding the cheeks, gums and tongue, and occurs through electrostatic attraction, hydrophobic interactions and hydrogen bonding. It is known that for clearance of food from the oral cavity, a low viscosity fluid works best (Chen & Engelen, 2012; Negoro et al., 2000). In the present study, the saliva from the LF group was not only relatively lower in volume, but also more viscous. Sufficient oral clearance may have been hindered in the LF group, leaving behind a higher quantity of dairy protein on the oral mucosa between swallows, and the greater perceptual drying sensation. In support of this theory, Norton et al. (2020) recently found higher amounts of mucoadhesion in a low saliva flow group after consumption of a whey protein beverage. In the same study, older adults were found to have significantly higher quantities of adhered protein post-whey beverage consumption. Older adults have also been found to be more sensitive to milk-protein elicited mouth-drying, compared to younger adults (Withers et al., 2013).

It must be noted that if a greater extent of mucoadhesion was present in the LF group, the participants were relatively unaware, as there was no significant difference in the perceptual intensity ratings of ‘mouthcoating’ between groups Fig. 4c. Though, the sensory standard used to train participants to recognise mouthcoating was full-fat cream (Table 1), which compared with the ONS in the current study, was relatively higher in fat but also relatively lower in protein. Fat in dairy products is also a source of mouthcoating (Aime, Arntfield, Malcolmson, & Ryland, 2001; Prinz, Huntjens, & De Wijk, 2006) and a focus group has previously defined ‘Fatty mouthcoat’ and ‘Dry lingering mouthcoat’ as separate sensations (Porubcan & Vickers, 2005). Therefore, for each product, perhaps the contribution made by each macronutrient to the oral coatings differed, and this resulted in different perceptual sensations, causing the discrepancies between findings.

A greater extent of mucoadhesion in the LF group could explain other sensory effects observed. The LF group perceived significantly higher aftertaste than the HF group (Fig. 4e). It is known that retro-nasal olfaction, along with gustation, can persist for a prolonged period after food has been swallowed (Buffo, Rapp, & Reineccius, 2005; Linforth & Taylor, 2000). This is likely due to a ‘reservoir’ of tastants and aroma volatiles within residual ONS which is absorbed on the mucosa lining the mouth and/or pharynx mucosa by mucoadhesive forces after swallowing (Buettner, Beer, Hannig, Settles, & Schieberle, 2002; Cook et al., 2017). Buttner et al. (2002) illustrated that between 30% and 40% of aroma compounds can be retained on the oral and pharyngeal mucosa. In fact, mucoadhesive polymers have been proposed as a method to prolong the residence time, and therefore perception, of flavour compounds on oral surfaces (Cook et al., 2017; Dinu et al., 2019, Dinu et al., 2019). In the same way the LF group may have been less able to clear the drying proteins from the mouth, flavour compounds are likely to have persisted to a greater extent in-between sips leading to the significantly higher aftertaste perception. These findings strengthen the crucial importance of ensuring adequate hydration is continuously accessible for patients with hyposalivation. Not only to fundamentally maintain adequate hydration status but also to enhance salivary flow and provide the opportunity to remove any lingering food flavours occurring post-consumption.

Significant differences in aroma release parameters (Tmax and Imax) were also found between groups. On average, the LF group reached Imax approximately 4 s later than the HF group (Fig. 8). According to Linforth, Baek, and Taylor (1999) Tmax of volatile release is usually defined by the moment of swallowing and therefore, the longer food remained in the mouth (slower eating) the greater the value for Tmax (Hollowood, 2002). Although we had aimed to standardise oral transit time in the present study, swallowing is a behaviour under both voluntary and reflex control (Ertekin et al., 2001) and is therefore challenging to control under experimental conditions. Aprea, Biasioli, Gasperi, Märk, and Van Ruth (2006) also found that participants who were asked to follow an oral processing protocol retained natural swallowing behaviour. Interestingly, reduced saliva flow has been found to increase oral transit time and reduce swallowing efficiency (Hughes et al., 1987; Rogus-Pulia et al., 2016). It could therefore be the case that, after placing in the mouth, the HF group swallowed the 15 mL portion of ONS quickly, and it was efficiently cleared from the oral cavity. Whereas the LF group, with less saliva to facilitate swallowing, experienced a prolonged oral and/or pharyngeal stage of swallow, and subsequently delayed aroma release. In support of this hypothesis, Blissett, Hort, and Taylor (2006) found positive correlations between Tmax and swallowing time. As far as the author is aware, the relationship of SFR and Tmax has not been found in human participants previously. Though, in a mathematical model developed to understand aroma release from liquid food products, Tmax was found to decrease upon increasing SFR (Harrison & Hills, 1997). In line with the current hypothesis, the authors attributed this to the flavour being more quickly removed and therefore unavailable for release into the headspace (Harrison & Hills, 1997).

Averaged across sips, intensity of aroma release in the LF group was almost 30% more intense than the HF group (Fig. 8). A negative relationship between salivary flow rate and intensity of in-mouth aroma release has been found previously in model mouth systems (Odake et al., 1998; Van Ruth, 2000). Recently, Yang et al. (2020) found that upon stimulation by capsaicin, saliva flow of participants increased by 75%, leading to a decrease in aroma release intensity in vivo. Yang et al. (2020) suggested that enhanced saliva production is likely to dilute aroma compounds in the mouth, so aroma release from the liquid matrix was reduced and therefore volatiles were less bioavailable in the nose. This is supported by Hollowood (2002), who explained that when considering volatile release from a liquid product, dilution reduces the aqueous phase concentration, and hence lowers the breath volatile concentration.

In-mouth interactions between volatiles and salivary components are also known to occur which may influence extent of aroma release, through binding or enzymatic conversion (Ployon et al., 2017). Pagès-Hélary et al. (2014) found that both mucin and α-amylase have the ability to retain aroma molecules within saliva, and these findings were recently supported by Dinu, Gadon, et al. (2019). A significantly greater PSR in the HF group, along with greater amylase activity, may have facilitated greater protein-aroma binding and therefore contributed to the relatively lower in-mouth aroma release in the HF group.

It could be anticipated that significantly higher aroma release intensity would drive differences in flavour perception, though no significant differences in ‘banana flavour intensity’ were found between groups. However, from viewing Fig. 4, we observe that the LF group reported a marginally higher perception of banana flavour at Sips 5 and 7. We hypothesise that with larger group sizes the significantly higher aroma release observed in the LF group would have translated into a significantly higher perception of banana flavour, thus this warrants further investigation.

All hedonic and subjective appetite measures had a significant (or borderline significant) effect of sip (see Appendix E) representing a decrease in enjoyment, and reduction in appetite, during ONS consumption. The decline in pleasantness ratings during ONS consumption agrees with previous findings on ONS consumption (Methven et al., 2010; Regan et al., 2019; Thomas et al., 2016). The first sip or taste of any food is typically the most pleasant and this effect can be explained by the effect sensory-specific satiation (SSS) or satiety. SSS is defined as the decline in wanting or liking of a food as it is eaten relative to uneaten foods during a single eating episode (Hetherington & Havermans, 2013; Nolan & Hetherington, 2009; Weijzen, Smeets, & De Graaf, 2009). Though not significant, averaged over the eight sips, the LF group rated pleasantness lower, compared with the HF group, by an average of 1.39 points on the scale (Fig. 5). It has been proposed that certain attributes which are disliked in ONS, such as mouth-drying and aftertaste, may contribute to the decline in liking over repeated consumption of ONS (Methven et al., 2010; Regan et al., 2019; Thomas et al., 2016). Hence, the greater perception of these attributes likely contributed to the lower hedonic ratings of the LF group. The increased feelings of fullness, and reduction in hunger, during ONS consumption has been found previously (Regan et al., 2019) and can be explained by the relatively high consumption of nutrients and energy within a full portion (125 mL) of ONS (Regan et al., 2019).

Compared with the HF group, we observed greater decreases in hunger ratings, for the LF and MF group (see Fig. 7a), which reached borderline significance (p = 0.08). In addition, though not significant, fullness ratings increased to a greater extent over the 8 sips for the LF group, compared with the MF and HF group. A wealth of data demonstrates how a more intense (Bolhuis, Lakemond, De Wijk, ; Ramaekers et al., 2014; Tang, Tan, Teo, & Forde, 2020; Weijzen, Zandstra, Alfieri, & De Graaf, 2008) or longer duration (De Graaf, 2012; Ramaekers et al., 2014; Ruijschop, Boelrijk, A. De Ru, De Graaf, & Westerterp-Plantenga, 2008; Tang et al., 2020; Zijlstra, De Wijk, Mars, Stafleu, & De Graaf, 2009; Zijlstra, Mars, Stafleu, & De Graaf, 2010) of flavour release and/or oro-sensory perception is known to suppress appetite and can lead to reduced food intake. As described by Yin, Hewson, Linforth, Taylor, and Fisk (2017) and Gibson and Brunstrom (2007) individuals may gradually learn that food with more intense or complex sensory profiles, may be more nutritionally rich, and therefore more satiating. It is a possibility that over increasing sips, more intense and prolonged aroma release and sensory perception, as a result of relatively low SFR, contributed to a greater satiation response in the LF group. These findings are important because, for patients with hyposalivation, a greater decrease in subjective appetite sensations during ONS consumption may contribute to premature termination of ONS intake.

### 4.1 Limitations

To alleviate the influence of medication and disease on sensory and flavour perception, the present study chose to recruit healthy adults as participants, rather than patients with hyposalivation. Therefore, our sample contained few individuals with a low saliva flow rate (n = 5), and therefore our study could be sensitive to the ‘small study effect’ phenomenon (Sterne, Gavaghan, & Egger, 2000). We therefore recommend findings are validated with larger group sizes to generate firm conclusions.

## 5 Conclusions

This study has found that group salivary differences are associated with variations in temporal sensory perception and in-mouth aroma release during consumption of a liquid ONS. Over repeated sips of ONS, a group with a low salivary flow rate, along with a low protein secretion rate, perceived greater aftertaste and a greater build-up of mouth-drying; we propose that this may have been caused by reduced oral clearance and a greater extent of mucoadhesion. This group also experienced relatively higher intensity of in-mouth aroma release likely to be caused by a lower in-mouth dilution of volatiles. These factors may have contributed to greater appetite suppression and reduced subjective ratings of pleasantness, through sensory-specific satiety. Due to small group sizes, further research should i) validate significant and borderline findings with larger group sizes in healthy individuals with hyposalivation and ii) validate findings in patients with hyposalivation. The unique sensory experience and preferences of individuals with hyposalivation should be considered, both clinically and in product development, to ensure food palatability and adequate nutritional intake. To improve the sensory experience, it may be helpful to provide ONS alongside saliva-stimulating interventions, to mitigate the undesirable temporal sensations perceived during consumption. In addition, these findings strengthen the crucial importance of ensuring adequate hydration is continuously accessible for patients with hyposalivation, to enhance salivary flow, and provide the opportunity to remove lingering flavour occurring post-consumption.

## Author contributions

All authors approved the final version of this article. Sophie Lester carried out the data collection. Sophie Lester, Ian Fisk, Moira Taylor, Leonardo Cornacchia, Marleen Kleijn, Katherine Hurst, Vlad Dinu and Charfedinne Ayed contributed to the conceptualisation, design of the project, interpretation of results and preparation of the manuscript.

## Ethics statement

This study was approved by Faculty of Medicine and Health Sciences Research Ethics Committee at the University of Nottingham (Reference No. 207–1902).

## Declaration of competing interest

Leonardo Cornacchia and Marleen Kleijn are employees of Danone Nutricia Research. All other authors have no interests to declare.

## Figures and Tables

**Table 5:** Mean, Standard error (SE), Minimum (min) and Maximum (max) values of each salivary constituent for each group classified by saliva flow rate. Significant differences between groups are indicated in both, letters indicate statistical group differences as determined by Tukey's test.

Group	Mean saliva flow rate (mL/min)	Mean saliva viscosity (mPas)	Mean saliva total protein concentration (mg/mL)	Protein secretion rate (mg/min)	Amylase activity (U/mL)
LF group					
Mean	**0.4**^**a**^	1.8	1.5	**0.6**^**a**^	45.0
SE	0.1	0.2	0.2	0.1	6.00
Min	0.3	1.3	1.0	0.4	24.0
Max	0.5	2.3	1.9	0.8	76.0
MF group					
Mean	**1.0**^**b**^	1.6	1.2	**1.1**^**b**^	50.0
SE	0.1	0.1	0.1	0.1	5.90
Min	0.7	1.0	0.7	0.6	2.00
Max	1.2	2.3	2.1	1.7	118
HF group					
Mean	**1.5**^**c**^	1.4	1.3	**1.9**^**c**^	57.0
SE	0.1	0.1	0.1	0.2	7.60
Min	1.3	0.9	0.8	1.3	18.0
Max	1.8	2.4	1.9	3.1	126
**ANOVAp value** **F statistic**	**<0.001** **58.166**	0.302 1.253	0.512 0.687	**<0.001** **17.725**	0.759 0.278

**Table 6:** Mean (±SE) intensity ratings for all sensory attributes, over four sips (sip 1, 3, 5 and 7), for each group.

	Sip 1	Sip 3	Sip 5	Sip 7
**Sweetness**				
Low flow group	5.84 (1.36)	5.74 (1.07)	6.34 (1.36)	6.76 (0.86)
Medium flow group	5.79 (0.43)	6.22 (0.31)	6.11 (0.38)	6.73 (0.42)
High flow group	5.16 (0.72)	5.64 (0.67)	5.53 (0.83)	5.41 (0.91)
**Banana flavour**				
Low flow group	6.02 (0.80)	6.08 (0.67)	6.78 (0.59)	7.56 (0.59)
Medium flow group	6.48 (0.40)	6.24 (0.34)	5.52 (0.60)	5.66 (0.45)
High flow group	6.23 (0.56)	6.10 (0.56)	6.04 (0.73)	5.84 (0.97)
**Mouthcoating**				
Low flow group	6.54 (0.60)	6.34 (0.86)	6.98 (0.64)	6.66 (0.98)
Medium flow group	5.41 (0.39)	5.21 (0.56)	6.26 (0.53)	6.18 (0.56)
High flow group	4.60 (0.77)	4.92 (0.80)	5.74 (0.98)	5.62 (1.02)
**Mouth-drying**				
Low flow group	4.32 (1.40)	5.04 (0.70)	6.60 (0.23)	7.72 (0.22)
Medium flow group	5.69 (0.51)	5.24 (0.55)	5.94 (0.60)	6.04 (0.52)
High flow group	5.43 (0.66)	4.67 (0.84)	4.80 (0.84)	4.66 (0.82)
**Aftertaste**				
Low flow group	7.64 (0.47)	6.80 (0.91)	8.36 (0.40)	8.76 (0.32)
Medium flow group	6.64 (0.54)	5.69 (0.51)	5.39 (0.58)	6.32 (0.56)
High flow group	5.47 (0.65)	4.68 (0.63)	5.09 (0.51)	5.13 (0.82)

**Table 7:** Mean Baseline and Change from baseline ratings (±SE), over sips 2, 4, 6 and 8, for all appetite variables, for each group

	Baseline	Change from baseline			
		Sip 2	Sip 4	Sip 6	Sip 8
**Hunger**					
Low flow group	6.42 (0.65)	−0.14 (0.30)	−0.38 (0.57)	−2.16 (1.14)	−2.60 (1.05)
Medium flow group	5.53 (0.56)	−0.57 (0.28)	−1.04 (0.41)	−1.75 (0.49)	−2.21 (0.61)
High flow group	4.90 (0.91)	0.24 (0.16)	0.24 (0.24)	0.00 (0.31)	−0.34 (0.45)
**Fullness**					
Low flow group	2.42 (0.64)	0.48 (0.24)	2.14 (1.12)	3.28 (1.24)	3.24 (0.96)
Medium flow group	3.69 (0.55)	0.62 (0.34)	1.34 (0.46)	1.88 (0.62)	1.18 (0.89)
High flow group	3.77 (0.78)	0.06 (0.21)	0.96 (0.40)	0.86 (0.40)	1.66 (0.38)
**Desire to eat**					
Low flow group	6.86 (0.44)	0.04 (0.20)	−0.80 (0.57)	−1.84 (0.78)	−2.28 (1.20)
Medium flow group	5.77 (0.70)	−0.56 (0.25)	−1.09 (0.35)	−1.89 (0.40)	−2.19 (0.59)
High flow group	5.80 (0.96)	−0.21 (0.31)	−0.51 (0.15)	−0.87 (0.21)	−1.79 (0.32)
**Prospective consumption**					
Low flow group	7.08 (0.54)	0.32 (0.27)	−0.88 (0.44)	−1.58 (0.30)	−1.70 (0.51)
Medium flow group	5.58 (0.51)	−0.24 (0.23)	−0.96 (0.35)	−1.32 (0.42)	−1.73 (0.52)
High flow group	5.19 (0.65)	−0.11 (0.31)	−0.31 (0.33)	−0.51 (0.65)	−1.20 (0.41)

**Table 8:** Mean (±SE) hedonic ratings, over sips 2, 4, 6 and 8, for each group.

	Sip 2	Sip 4	Sip 6	Sip 8
**Pleasantness**				
Low flow group	4.92 (1.00)	5.02 (1.32)	4.04 (1.46)	4.02 (1.45)
Medium flow group	6.01 (0.39)	5.79 (0.46)	5.60 (0.53)	4.43 (0.57)
High flow group	5.92 (0.38)	6.10 (0.42)	6.14 (0.47)	5.40 (0.81)
**Desire to drink more**				
Low flow group	4.58 (1.55)	3.92 (1.61)	3.58 (1.61)	3.88 (1.71)
Medium flow group	4.95 (0.62)	4.71 (0.59)	4.88 (0.65)	3.85 (0.62)
High flow group	5.03 (0.85)	5.67 (0.89)	4.73 (0.97)	4.21 (0.87)

**Table 9:** Main effects and pairwise comparisons (Tukey) for each Sip effect of appetite variables as analysed by Mixed models ANOVA. Significant differences are highlighted in bold.

		Beverage pleasantness	Hunger	Fullness	Desire to eat	Prospective consumption	Desire to drink more of beverage
**Main effect of Sip**							
p-value F statistic DFn, DFd		0.057 3.049 1.964, 53.03	**<0.001** 14.17 1.450, 39.16	**0.004** 6.247 2.026, 54,71	**<0.001** 18.10 1.506, 40.65	**<0.001** 15.06 1.957, 52.84	**0.047** 3.098 2.231,60.23
**p-values as determined by pairwise comparisons (Tukey)**							
Sip 2	Sip 4 Sip 6 Sip 8	0.996 0.654 **0.047**	0.330 **0.009** **0.002**	**0.011** **0.002** 0.107	0.054 **<0.001** **<0.001**	**0.006** **0.003** **<0.001**	0.994 0.831 **0.016**
Sip 4	Sip 2 Sip 6 Sip 8	0.996 0.758 **0.041**	0.330 **0.007** **0.001**	**0.011** 0.328 0.895	0.054 **<0.001** **<0.001**	**0.006** 0.229 **<0.001**	0.994 0.848 **0.003**
Sip 6	Sip 2 Sip 4 Sip 8	0.654 0.758 0.231	**0.009** **0.007** **0.032**	**0.002** 0.328 0.989	**<0.001** **<0.001** 0.059	**0.003** 0.229 0.049	0.831 0.848 0.195
Sip 8	Sip 2 Sip 4 Sip 6	**0.047** **0.041** 0.231	**0.002** **0.001** **0.032**	0.107 0.895 0.989	**<0.001** **<0.001** 0.059	**<0.001** **<0.001** 0.049	**0.016** **0.003** 0.195
